# Saddle point localization of molecular wavefunctions

**DOI:** 10.1038/srep33068

**Published:** 2016-09-15

**Authors:** Georg Ch. Mellau, Alexandra A. Kyuberis, Oleg L. Polyansky, Nikolai Zobov, Robert W. Field

**Affiliations:** 1 Physikalisch-Chemisches Institut, Justus-Liebig-Universität Giessen, Heinrich-Buff-Ring 17, D-35392 Giessen, Germany; 2 Institute of Applied Physics, Russian Academy of Science, 46 Uljanov Street, Nizhny Novgorod, Russia; 3 Department of Physics and Astronomy, University College London, Gower St, London, UK; 4 Department of Chemistry, Massachusetts Institute of Technology, Cambridge, Massachusetts 02139, USA

## Abstract

The quantum mechanical description of isomerization is based on bound eigenstates of the molecular potential energy surface. For the near-minimum regions there is a textbook-based relationship between the potential and eigenenergies. Here we show how the saddle point region that connects the two minima is encoded in the eigenstates of the model quartic potential and in the energy levels of the [H, C, N] potential energy surface. We model the spacing of the eigenenergies with the energy dependent classical oscillation frequency decreasing to zero at the saddle point. The eigenstates with the smallest spacing are localized at the saddle point. The analysis of the HCN ↔ HNC isomerization states shows that the eigenstates with small energy spacing relative to the effective (*v*_1_, *v*_3_, *ℓ*) bending potentials are highly localized in the bending coordinate at the transition state. These spectroscopically detectable states represent a chemical marker of the transition state in the eigenenergy spectrum. The method developed here provides a basis for modeling characteristic patterns in the eigenenergy spectrum of bound states.

The design of experimental methods based only on first principles to measure chemical properties is an important research topic of physical chemistry. Isomerization reactions take place within the bound energy region of a molecular system; the measurement of molecular eigenenergies is such a first principles related experimental method. It does not follow the usual predict, measure, and compare methodology: the multi-dimensional eigenvalue equation of the molecular system is extremely simple to formulate but it is impossible to solve^1^,^2^. In high-resolution spectroscopy we use experiments to “solve” the exact molecular Schrödinger equation^3^ and correlate the experimental results with potential energy surface(PES) based models.

Classical spectroscopic experiments collect data that mainly sample only one type of stationary point on the PES: local minima. These minima describe the isomers of the molecular system and define the concept of the molecular structure. For example, the PES of the [H,C,N] molecular system formed from the atoms C, N and H has two minima corresponding to the molecules HCN and HNC. Between the collected spectroscopic information and the near-minimum region of the potential there is a textbook-based relationship. In this way a complete set of molecular eigenenergies collected at low molecular excitations^4^,^5^,^6^ is equivalent to the near-minimum region of the potential. The eigenstate-based description can be transformed to a time domain description^7^,^8^ giving us important insights into the molecular dynamics of the molecular system. Whichever description we use, to be “chemistry dynamics relevant” it is necessary to measure, calculate and interpret the highly excited molecular rovibrational eigenstates. We can assign spectroscopic experiments if we can identify specific spectroscopic patterns.

The PES of a molecule has another type of stationary points that is very important in chemistry: the saddle points. Saddle points correspond to chemical processes through transition state theory. In contrast to the near-minimum regions, standard textbooks provide no guidance how the potential connecting the two minima is encoded in the eigenstates^6^,^9^. In this work, we show how one can detect special eigenstates in the measured eigenenergy spectrum that sample the spatial region of the PES saddle point and that possess unique features: localization at the saddle point and exhibiting a minimum eigenenergy spacing. These special first principle based transition state levels directly correlate with the transition state concept of chemistry.

## Correspondence principle and molecular dynamics in the frequency domain

The correspondence principle^10^ of quantum mechanics is not only a rule that any quantum mechanical theory we set up must satisfy, but has fundamental importance in interpreting any quantum mechanical result we obtain. The consequence of this principle is the possibility to connect the quantum description to the corresponding classical one in some region of system parameters. One such classical to quantum mechanical correspondence is the correlation between the spacing of the discrete eigenenergies and the classical oscillation frequency in a bound system. The classical oscillation frequency *ω*_*c*_(*E*) times *ħ* at the total excitation energy *E* must match approximately the energy spacing 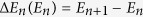 of the quantum states


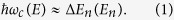


For systems where this correlation is valid we can gain a physical insight regarding the structure of the energy eigenvalues for a bound quantum system based on purely classical calculations. There are only a few quantum models where analytical solutions for the energy eigenvalues can be given. The correspondence principle allows us to study systems where there is no analytical solution for the quantum problem but where we can find an analytical solution for the classical problem.

To obtain a visualization of the quantum dynamics in the frequency domain we can represent the spacing of a set of eigenenergies Δ*E*_*n*_(*E*_*n*_) and compare it with the result obtained in the classical limit. In any region of the classical phase space where dramatic changes in the system dynamics take place, there will be a significant change in the corresponding Δ*E*_*n*_(*E*_*n*_) quantum frequency. We expect that these Δ*E*_*n*_ curves show specific quantum signatures.

There are two physical quantities involved here: *ħω*_*c*_(*E*) is the classical frequency scaled with *ħ* and Δ*E*_*n*_(*E*_*n*_) is the quantum frequency. For practical reasons we consider *ħω*_*c*_ as the classical frequency and use energy related dimensions to describe both frequencies. In the Supplementary Information we show that for the Morse^11^ potential there is an exact classical to quantum mechanical correspondence *ħω*_*c*_(*E*) = Δ*E*_*n*_(*E*_*n*_) if we consider the following correspondence between the quantum mechanical eigenenergies *E*_*n*_ and classical total energy *E*


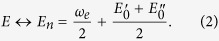


Even if the Morse potential is a fundamentally anharmonic potential the calibration of the mean spectroscopic eigenenergies to the classical oscillation frequencies must be performed based on the *ω*_*e*_ harmonic frequency. This is a very intuitive result: The zero point energy correction must be evaluated according to the small amplitude classical oscillation frequency.

The symmetric double-well quartic potential^12^,^13^,^14^


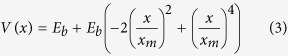


has a saddle point between two symmetric minima; for this potential an analytic solution of the classical motion^12^ exists. The classical oscillation frequency for *E* < *E*_*b*_, where *E*_*b*_ is the barrier height, is given as


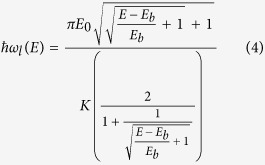


and for *E* > *E*_*b*_ it is


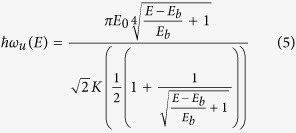


where *K*(*k*) is the normal elliptic integral of the first kind. For small energy *E* the oscillations for a system with mass *m* are harmonic with the frequency *ω*_0_ around the positions centered at *x* = ±*x*_*m*_. To compare the classical and quantum frequencies we factored out 
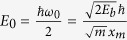
, the ground state eigenenergy of the quantum system which corresponds to the classical small amplitude harmonic oscillations.

**Figure 1:**
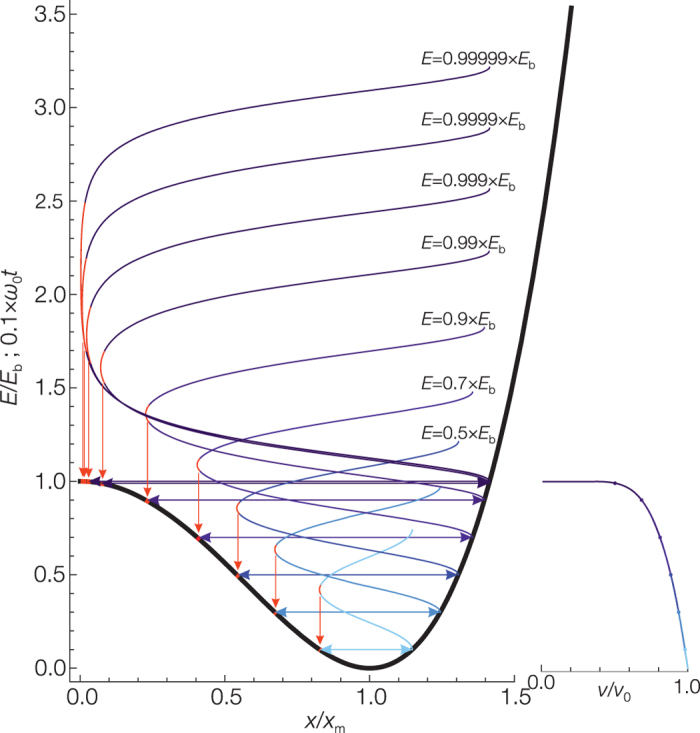
The *x* > 0 portion of the symmetric double-well potential *E*_*b*_ + *E*_*b*_(−2 × (*x*/*x*_*m*_)^2^ + (*x*/*x*_*m*_)^4^), the classical trajectories for selected energies *E* and the energy dependence of the oscillation frequency *ν*. The trajectory section marked in red at the saddle point near turning point corresponds to 1% of the region between the turning points, *ν*_0_ is the small amplitude harmonic oscillation frequency. As the excitation energy approaches the barrier height *E*_*b*_ the system spends a larger and larger fraction of the oscillation period in the neighborhood of the saddle point. The increasing spatial localization at the saddle point correlates with the decrease to zero of the oscillation frequency. This *E* → *E*_*b*_ limit corresponds to the concept of instanton.

Figure 1 shows the potential for *x* > 0 and the exact classical trajectories as the energy asymptotically approaches the top of the barrier. As the excitation energy converges to the barrier height *E*_*b*_ the system spends a larger fraction of the oscillation period in the neighborhood of the saddle point. The increasing spatial localization at the saddle point correlates with the decrease to zero of the oscillation frequency.

**Figure 2:**
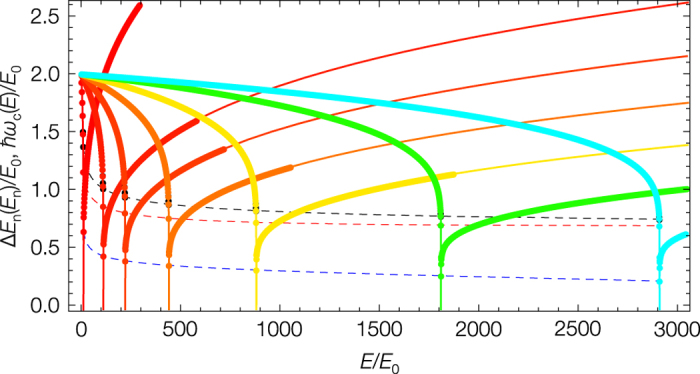
The plot of the classical oscillation frequency *ħω*_*c*_(*E*) and the corresponding discrete quantum frequencies Δ*E*_*n*_(*E*_*n*_) for the symmetric double-well quartic potential shown in Fig. 1. The curves correspond to potentials with increasing barrier heights *E*_*b*_ = *i* × *E*_0_ with *i* = 11 (red for even states and black for overlapping odd states), 111, 221, 441, 881, 1811, 2911(blue). *E*_0_ is the harmonic zero point energy and Δ*E*_*n*_ is represented using Equation 2. The dashed curve connects the quantum frequencies below (red and black curve) and above the saddle point (blue curve). Below the barrier there are two overlapping sets of quantum frequencies corresponding to the classical oscillations in the two wells. The analytical solution for the classical oscillation frequency models the eigenenergy spacing including the frequency dip in the neighborhood of the saddle point of the potential.

**Figure 3:**
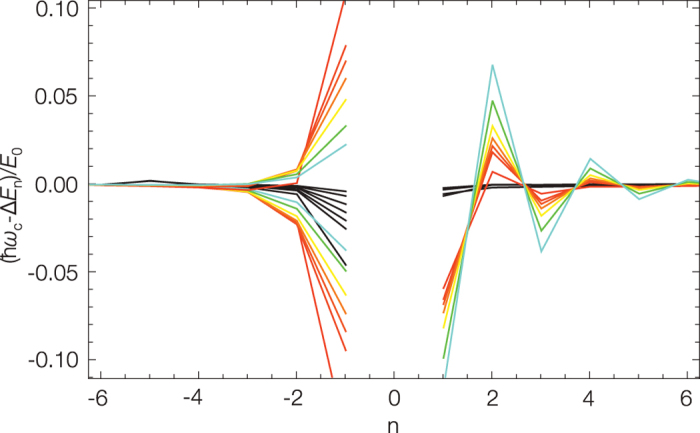
The difference between the classical and quantum frequencies in the neighborhood of the saddle point for the symmetric double-well quartic potentials with increasing barrier heights as defined in Fig. 2. The red to blue colour code corresponds to increasing barrier heights, black curves show the semiclassical case; *n* > 0 counts the quantum frequencies above and *n* < 0 below the saddle point. Below the barrier the curves with a negative value correspond to the even states. The eigenenergy spacing of the states at the saddle point region stops following the classical frequency with the same accuracy as it does well below and above the barrier. The difference between the classical and quantum frequencies below (the pattern of the tunnel effect) and above the saddle point show typical patterns; the increase of the frequency difference correlates with the increasing localization of the wavefunctions at the saddle point.

We can study the classical to quantum correspondence (1) by increasing the barrier height *E*_*b*_ and thereby tuning the position of the eigenstates to the neighborhood of the barrier. The classical frequencies for potentials with increasing barrier heights *E*_*b*_ = *i* × *E*_0_ with *i* = 11, 111, 221, 441, 881, 1811, 2911 and the corresponding quantum frequencies calculated using the Fourier grid Hamiltonian method^15^ are shown in Fig. 2; here we use the same energy correlation 2. The analytical solution for the classical oscillation frequency models the eigenenergy spacing including the frequency dip in the neighborhood of the saddle point. Figure 3 shows the difference between the classical and quantum frequencies. The quantum system must avoid touching the barrier; even in the case of large quantum numbers there remains a difference between the classical and quantum frequencies in the neighborhood of the saddle point.

It is possible to set up an analytical model for the eigenenergy spectrum for the quartic potential when the eigenenergies are known


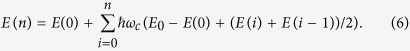


This model based on the classical frequency *ħω*_*c*_(*E*) allows adjustment of the experimental data and obtain a fit for the *E*_*b*_ parameter of the quantum mechanical system.

## Molecular dynamics at the isomerization barrier: The eigenstate perspective

Rovibrational molecular spectroscopy is based on the existence of eigenenergy patterns related to the internal molecular dynamics. We see these patterns in high resolution spectra and they represent our “spectroscopic view” of the molecular system. One special type of pattern we expect to see in molecular spectra is related to the existence of a saddle point on the potential energy surface.

The [H,C,N] molecular system is an ideal candidate to study the eigenstates in the neighborhood of a saddle point. The saddle point corresponds to the transition state of the simple HCN ↔ HNC hydrogen shift isomerization reaction between the HCN and HNC linear isomers. In the isomerization reaction the *ν*_2_ bending mode is coupled to the *ν*_1_ HC/HN stretch mode and represents the reaction coordinate. The CN stretch vibration is a spectator mode only slightly coupled to the isomerization.

For molecules the *E*_*n*_ eigenenergies correspond to the wavenumbers of the rovibrational eigenstates and the *ω*_*c*_ frequency of the classical oscillations corresponds to the classical motion in the molecular Born-Oppenheimer potential energy surface. Correlation (1) has important consequences regarding the energy eigenstates of molecules. To study it for a polyatomic molecule a complete set of assigned energy eigenstates, *E*_*n*_ up to very high excitation energies is needed.

**Figure 4:**
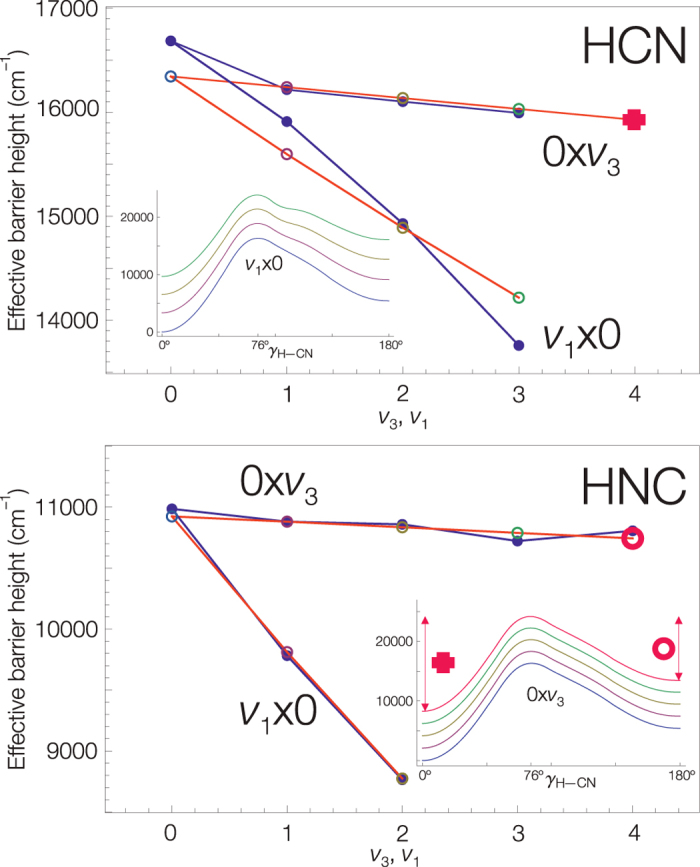
The H-C/H-N and C-N stretch excitation dependence on the (*ν*_1_, *ν*_3_, *ℓ* = 0) dynamic isomerization barrier heights for HCN and HNC. The data points joined with red lines correspond to the barrier heights of the 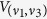 bending pseudopotentials calculated by Joyeux *et al*.^19^; the potentials for the (*ν*_1_, 0) and (0, *ν*_3_) are shown as inlets. The data points joined with blue lines are the barrier heights determined from the eigenenergy based quantum frequency correspondence analysis. The upper curve corresponds to C-N stretch spectator excitation; the barrier height is practically constant. The lower curve corresponds to H-C/H-N stretch excitation coupled with the bend isomerization mode. The two barrier heights agree within 1–2%, this agreement allows us to define pseudopotentials from pure spectroscopic experiments. The transition state can thus be considered a well defined pure quantum mechanical concept defined through molecular eigenstates.

The basic concepts of the 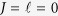 internal dynamics for the [H,C,N] molecular system have been developed by Bacic and Light^16^,^17^. They show that the onset of delocalization is governed by the height of the effective adiabatic-bend potential barrier. For each 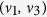 stretch combination they define an effective dynamic bending potential. Such dynamic potentials have been calculated for the [H,C,N] global potential^18^ used in this work by Joyeux *et al*.^19^ and are shown in Fig. 4.

**Figure 5:**
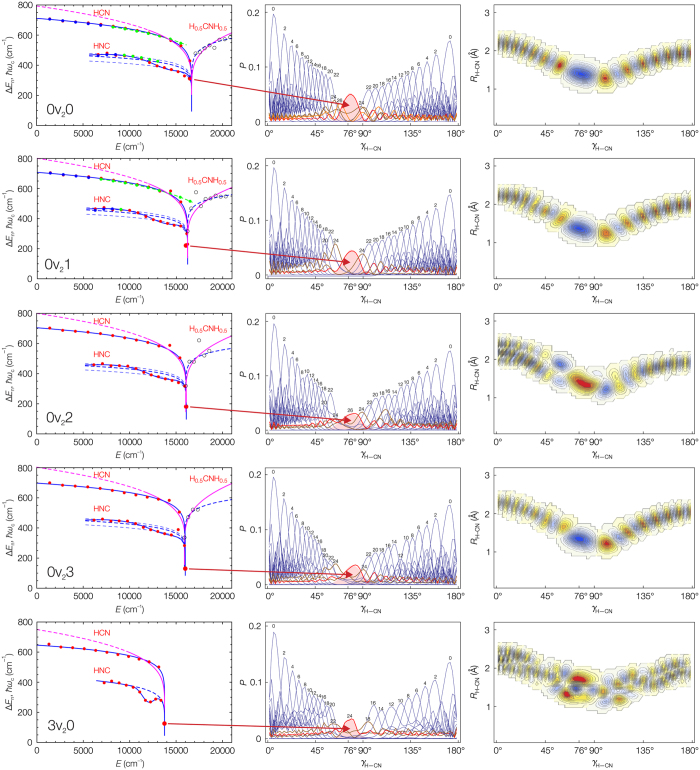
The emergence of the *J* = 0 saddle point localized states for the [H,C,N] molecular system. Left curves: the quantum frequencies for the bending series of states (red: *ab initio* data, blue: measured data, green: predicted from measurements) and the analytical models (blue: Equation 7, magenta: Equations 4 and 5). Middle panels: the one-dimensional projection of the wavefunctions on the bending coordinate (red surface: the fitted minimum Gaussian wavepacket probability density curve). Right panels: the two dimensional projection of the wavefunctions for the saddle point localized states. The one dimensional projections show how the harmonic type, single-well bending wavefunctions of HCN and HNC converge to a final saddle point localized state; these states are strongly spatially localized at the *γ*_H–CN_ = 76° transition state. These states correspond to the eigenstates with the smallest quantum frequency and are thus detectable in a spectroscopic experiment. The frequently used two-dimensional representation includes the intensity of the ground state H-CN stretch excitation and shows the localization effect less clearly.

The first column of curves in Fig. 5 shows the bending quantum frequencies for HCN, HNC and above the barrier H_0.5_CNH_0.5_^3^ bond-breaking^20^ states for a selected constant set of 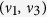 stretch excitations. For these series of states the corresponding classical oscillations take place due to the effective adiabatic-bend potential. The bending states climb the isomerization barrier in a potential well with a saddle point qualitatively similar to our model potential shown in Fig. 1. As expected, the [H,C,N] bending quantum frequency curves are similar to the quantum frequency curves shown in Fig. 2.

To model the bending quantum frequency curves we can try to set up simple analytical models. Considering the correspondence principle the general method to obtain analytical models for quantum frequency curves is to solve the oscillation frequency of the classical problem analytically. In general the quantum frequencies have different excitation energy dependence because the effective potentials differ for each dynamical isomerization path and molecule. This means that we can expect to find only approximate models. On the other hand even approximate analytical models are still very important. They can be used to set up the effective spectroscopic Hamiltonian in the energy region of the saddle point; this Hamiltonian represents the internal dynamics in the saddle point region of the phase space.

Baraban *et al*.^9^ studied the semiclassical pattern of eigenenergies at the saddle point based on the concept of the effective frequency 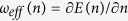. The knowledge of the analytical form *E*(*n*) of the eigenenergies for a one-dimensional potential allows to obtain the analytical model for the quantum number dependence of the effective frequency. In the absence of such a formula for a potential with a saddle point region they propose an ansatz type effective frequency formula expressed in the excitation energy. The formula is based on the analysis of the physical mechanism of the diatomic potentials and their typical effective frequency dependence. In this work, we use this as an ansatz type quantum frequency formula in a slightly changed form


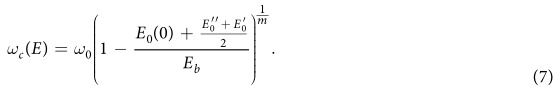


where we included the classical to quantum frequency correspondence 2; 
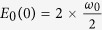
 is the zero level energy calculated for the twofold degenerate pure harmonic bending oscillations. For a simple comparison with spectroscopic parameters of the fit results we adjusted the model to the quantum frequencies divided by two. We used the analytical solutions (4) and (5) with the classical to quantum correspondence 2 as a second model for the functional form of the quantum frequency in the neighborhood of the saddle point.

The three-parameter dependent ansatz type model has been tested^9^ with an acetylene experimental data set containing four data points, and with a slightly extended experimental and ab initio HCN/HNC data set reported previously^6^. Our analysis of the HCN and HNC eigenenergy data shows a model precision between a few wavenumbers and a few tens of wavenumber for at least twenty pure bending and bending plus CN stretch quantum frequency series. For the bending plus CH stretch eigenenergies, this formula is less suitable to describe the quantum frequencies in the saddle point region. The two parameter dependent quantum frequency model (4) combined with the correspondence relation 2 is globally a less precise model for HCN and HNC but gives the potential related quantum frequency dependence at the saddle point. This model can be extended with additional parameters, this improves significantly the model precision but destroys the correspondence between the analytical form of the potential and the quantum frequency.

The quantum frequency curves fitted with the analytical models are shown in Fig. 5, the fitted model parameters are listen in Table S1. The quantum frequency analysis of the barrier-proximal^9^ vibrational levels allows extraction of the dynamic isomerization barrier heights from the eigenenergies alone. The 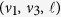 HCN and HNC dynamic barrier heights determined in the quantum frequency analysis agree within 1–2% with the pseudopotential barrier heights. The comparison of the barrier heights is displayed in Table S2 and is illustrated in Fig. 4. Figure 4 shows the dependence of the isomerization barrier height on increasing simultaneous stretch excitations. A significant decrease of the barrier height results only from excitation of the H-C or H-N stretch vibration.

As the pure bending eigenstates pass through the energy of the isomerization barrier, the hydrogen atom no longer “belongs” to one of the ends of the CN fragment. These vibrational states are extremely important for chemistry as they correspond to a third type of molecule that we can form from the H, C and N atoms: H_0.5_CNH_0.5_^3^. For this molecule there is no corresponding minimum on the molecular PES, we can correlate it with the saddle point. The chemical properties of a molecular gas with a significant number of molecules in the bond-breaking states differs from the chemical properties of a pure HCN or HNC molecular gas.

The theoretical description within the adiabatic approximation of bond-breaking states is based on the assumption that the stretch quantum numbers remain well-defined for bond-breaking + stretch mixed states. This is not a straightforward result and the validity of this model still requires an experimental verification. We assigned all states -without exception- to one of the bond-breaking + stretch eigenenergy progressions based on visual inspection of the wavefunctions. The majority of these states are strongly mixed from the viewpoint of the eigenstates below the barrier.

Figure S5 shows the bond-breaking quantum frequency curves for different 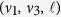 excitations, Table S3 gives the results of the analysis based on the models 7 and 5. These results emphasize the importance of the quantum frequency analysis; in fact this figure proves that in zero order, all stretch excitations remain well-defined up to very high excitation energies. For each 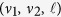 series the quantum frequency dependence is similar to the one obtained for the model potential shown in Fig. 2.

## Localization of molecular wavefunctions at the dynamic saddle points

The quantum frequency curves shown in Fig. 5 allow us to extract an extremely important piece of information for an eigenstate even if we do not know the effective potential: how close the eigenstate is to the top of the isomerization barrier. A careful study of the barrier-proximal state vectors allowed us to understand how the wavefunction of an eigenstate correlates with its distance to the isomerization barrier and what is the structure of the molecular wavefunctions in the neighborhood of the saddle point. The second column of panels in Fig. 5 shows the probability distribution *P* along the bending coordinate for the HCN and HNC molecular wavefunction for some 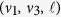 bending series. The one-dimensional projections clearly show how the harmonic type, single-well wavefunctions of HCN and HNC converge to a final state that is the one nearest to the saddle point. These eigenstates are spatially localized to a very high extent exactly at the *γ*_H–CN_ = 76° transition state. The eigenenergies of these states give the position of the 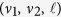 dynamic isomerization barriers to a high precision.

**Figure 6:**
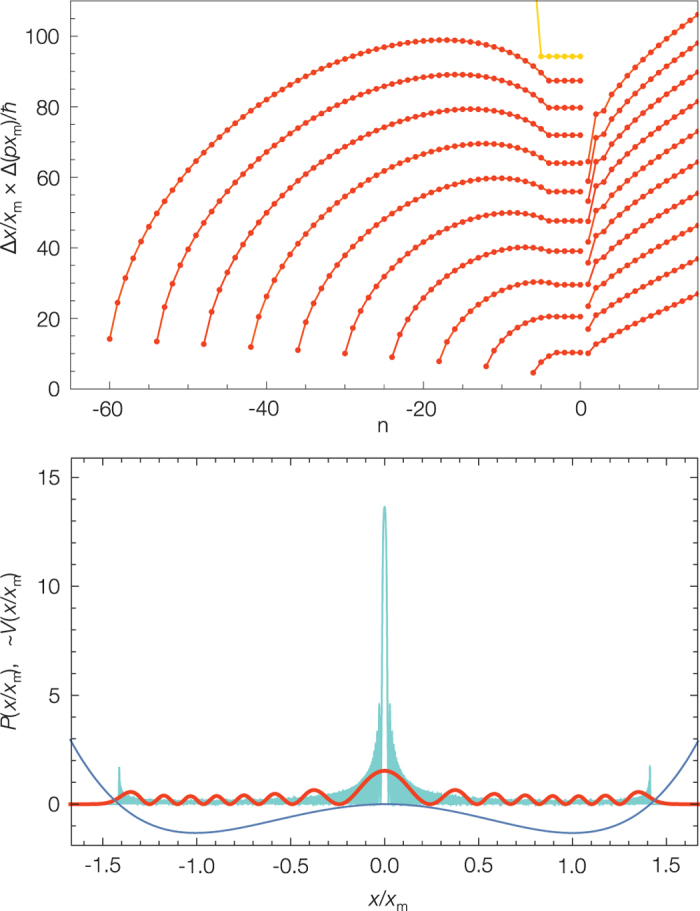
Upper panel: The quantum number dependence of the localization factor Δ*x* × Δ*p* for the symmetric double-well quartic potential with increasing *E*_*b*_ = *i* × *E*_0_ barrier heights for *i* = 1 + 10 × *j*, *j* = 1 … 10 (red) and for *i* = 811 (orange, shifted down); *n* > 0 counts the eigenstates above and n ≤ 0 below the saddle point. These curves show the quantum localization effect of the potential saddle point. Lower panel: The localization probability *P* for the eigenstate above the barrier for *i* = 11(red) and *i* = 2911. The wavefunctions of this one-dimensional model potential resembles the localization features of the bending part of the full dimensional molecular wavefunctions.

**Figure 7:**
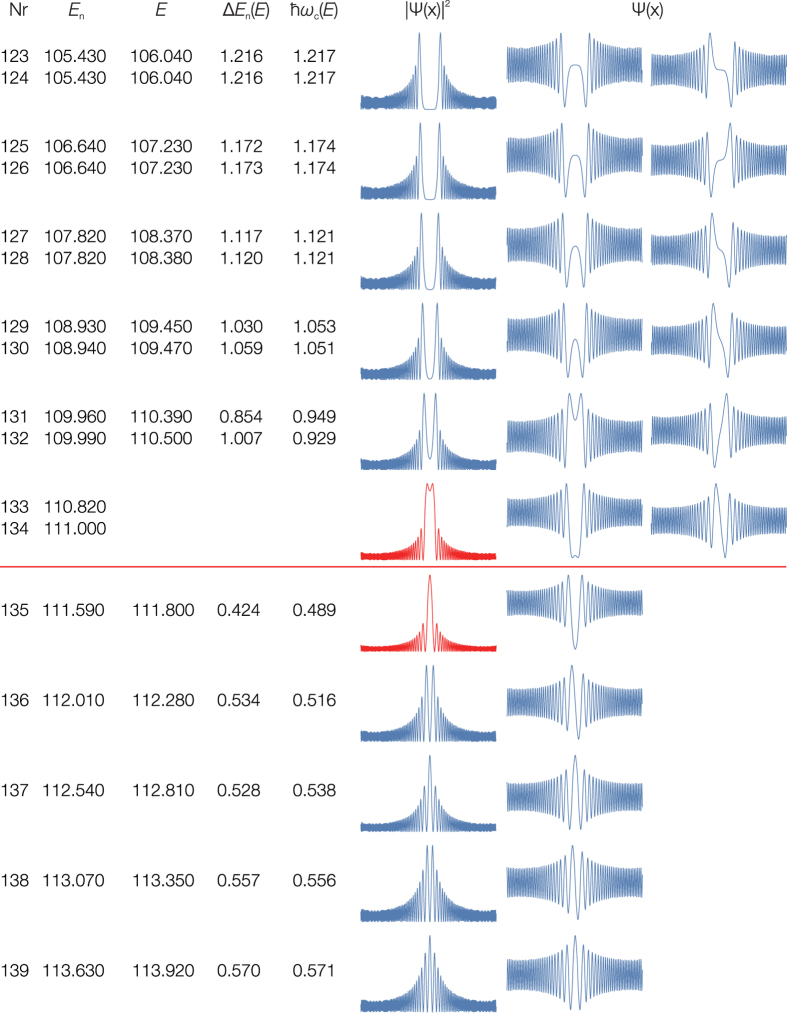
The quantum frequency analysis of the states in the neighborhood of the saddle point for the symmetric double-well quartic potential with the *E*_*b*_ = 111 × *E*_0_ barrier height. The eigenenergy of the nearest state to the barrier has the value *E*_*b*_ = 111.000 × *E*_0_ matching the barrier height. This is an example for the barrier avoidance, this takes place for the quantum frequencies and not for the eigenenergy values.

The localization at the saddle point in the case of the [H,C,N] molecular system does not occur by chance, the localization is a general feature of the eigenenergy spectrum of any system with saddle points. Figures 6,7 and S5 show the emergence of the barrier-localized eigenstates for the quartic potential. The wavefunctions of the barrier-proximal eigenstates with the energy close to that of the barrier overlap significantly with the saddle point itself.

The [H,C,N] molecular wavefunctions near the 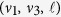 dynamic saddle points are localized similarly to this model. The emergence of the localized states at the saddle points of the effective adiabatic-bend potentials are shown for the 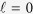 states in Fig. 5 and for 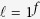 states in Figure S8. Figure S9 displays bending series where no clear saddle point localized states can be found. Either these states mix with other states and lose the localization character or there are simply no eigenstates near the barrier. In the latter case, the quantum frequencies of all states are larger than the ones found for saddle point localized states.

The spatial localization is a general feature of wavefunctions in the neighborhood of saddle points and can be intuitively understood based on the classical trajectories shown in Fig. 1. For oscillation energies near the barrier the classical system slows down and at the same time is highly localized. Such dynamically localized states are not allowed for a quantum system but a different quantum type localization still persists. The energy eigenstates corresponding to the saddle point localized trajectories are not eigenstates of the observables *x* and *p*. A measurement of the position or momentum can yield any result however the multiplicative factor of the root mean square deviations 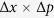 still measures the degree of quantum localization. For the harmonic oscillator this factor has for the ground state the minimum allowed value by the uncertainty relation and increases linearly with the quantum number. Figure 6 shows how the existence of a saddle point interrupts this linear increase, forcing the quantum system to become highly localized in comparison with a potential without saddle points.

Even if this quantum localization is a very fundamental and general property of the energy eigenstates, it seems that their importance and emergence have not been previously recognized. To our knowledge the localization of the eigenstates at the potential saddle point has been mentioned in two works: in the work of Dutta and Bhattacharyya^21^ regarding the eigenenergies of the one dimensional model potential and in the work of Henderson, Lam, and Tennyson^22^ regarding the KCN molecular wavefunctions at the barrier to linearity. Both papers report the existence of the barrier localized wavefunctions but do not explain the dynamical emergence of the localized wavefunctions and their connection to the eigenenergy spectrum as presented in this work.

The functional form of the localization probability at the saddle point for these wavefunctions corresponds to a minimum Gaussian wavepacket (see Figs 5 and S8).

Another important property of these states is the existence of a minimum quantum frequency as expected from a semiclassical viewpoint. Figure 2 shows the smallest quantum frequencies for the quartic model potential which correlate with barrier localized states. For the [H,C,N] molecular system, this corresponds to approximately *ω*_*loc*_ = 150 cm^−1^. This means that the position of the bending Q-branches that decrease in wavenumber as *v*_2_ increases in a HCN *ν*_2_ wavenumber region spectrum will be limited to a minimum of 150 cm^−1^. We expect to see the existence of this limit at low wavenumbers in molecular spectra as the unique spectroscopic pattern of a saddle point localized eigenstate.

The form of the *J* = 0 saddle point localized wavefunctions suggests that we may assign these states to any of the HCN or the HNC molecule. Indeed, from the point of view of the internal dynamics this state seems to be “degenerated”. The effective potential barrier heights match the barrier heights of the quantum frequency analysis (see Fig. 4) only if the eigenenergy of the saddle point localized state is taken into account simultaneously for HCN and HNC quantum frequency series (see Supplementary Information).

## Discussion and Outlook

The chemical structure of all known and still unknown molecules is encoded in the solutions of the formally simple time independent molecular Schrödinger equation. Exact numerical solutions of this equation are impossible to compute even for the simplest systems. High resolution molecular spectroscopy is the single way out of this dilemma; from spectroscopic measurements we can obtain the complete and very accurate set of eigenvalues^5^,^6^. However, the chemical information encoded in this data is useless for chemistry; we must interpret the spectroscopic information and convert it to the language of chemists.

In this work, we pointed out how the classical to quantum correspondence identifies characteristic patterns in the eigenenergy spectrum of bound states and we have shown that these patterns can be connected in a direct way to chemical concepts. We detected and explained the emergence of a very special set of saddle point localized eigenstates in the molecular energy spectrum. These special states are “chemical markers” in the eigenenergy list. Based on the correlation between classical and quantum frequency we pointed out a method to search for approximate analytical functional forms hat connect the eigenenergies of highly excited molecular states. These concepts allow the development of new zero order theoretical descriptions of the highly excited states.

The existence of the saddle point localized states has important implications regarding molecular spectroscopy as an experimental tool in chemistry. These states, when detected in molecular spectra, define the position of the transition states by themselves.

The frequency analysis presented in this work allows to study the internal dynamics of complex molecular systems. Quantum frequencies can be calculated for any complex system with any dimension: each molecule has a global quantum frequency network (QFN). For each molecule, the eigenenergies and transitions between the eigenstates define a complex spectroscopic network^23^. The eigenenergy nodes of this network can be transformed to a set of corresponding quantum frequency nodes. The frequency analysis for a complex system is the correlation of the QFN with the corresponding complex classical motion. The frequency analysis presented in this work correlates some specific paths of QFN with the classical motion in one-dimensional effective potentials. In this way, paths and sub-networks of the QFN with specific well-defined patterns correspond to regions of the phase space with interesting internal dynamics of the system. The procedure based on the QFN outlined above does not require knowledge of any of the approximate quantum numbers.

## Methods

The analysis of the [H,C,N] molecular system presented in this work is based on two complete rovibrational eigenenergy data sets that one of the authors developed over the last fifteen years. The first is based on experimental eigenenergies determined through the spectroscopy of hot molecular gases^3^,^4^,^5^,^6^,^24^,^25^,^26^,^27^. This data set is the most extensive and partially the most complete eigenenergy spectrum determined so far for a polyatomic molecule. To compare the HCN ab initio quantum frequencies with experimental ones a new Dunham type spectroscopic Hamiltonian has been calculated similar to the one reported for HNC in reference^27^. Additional to the vibrational states reported so far we included in this analysis the vibrational constants of highly excited *v*_2_ = 12, 13, 14 states determined form the newly assigned P,Q and R bands of a HCN emission spectrum in the pure bending region. The spectroscopic constants of these states are given in Table S5 and the vibrational expansion constants are listed in Table S6. The details of the analysis and of the models used can be found in the references^6^,^27^.

The second data set is the list of the complete, full dimensional Born-Oppenheimer rovibrational eigenenergies^28^,^29^,^30^,^31^ up to 1500 cm^−1^ above the isomerization barrier. This *ab initio* spectrum^18^,^32^,^33^ has been vibrationally assigned by Mellau^6^,^9^ based on the organization of the eigenenergies in regular patterns. For this work we extended the eigenenergy list up to 6000 cm^−1^ above the saddle point and stored the complete set of the state vectors. Our calculation improved on the previously reported^6^,^9^,^33^ not fully converged eigenenergies of the delocalized states by a few wavenumbers. In this work the pattern based eigenenergy analysis has been correlated with the analysis of the state vectors. We could confirm the early pattern based assignments and assign all states of the new extended data set.

## Additional Information

**How to cite this article**: Mellau, G. C. *et al*. Saddle point localization of molecular wavefunctions. *Sci. Rep.* **6**, 33068; doi: 10.1038/srep33068 (2016).

## Supplementary Material

Supplementary Information
