# Supplementary material for: Saddle point localization of molecular wavefunctions
Source: Sci Rep. 2016 Sep 15;6:33068. doi: 10.1038/srep33068 (PMC5024097; doi:10.1038/srep33068)
Supplement: Supplementary Information [file srep33068-s1.pdf]

Supplementary Information

Saddle point localization of molecular wavefunctions

Georg Ch. Mellau[1,*], Alexandra A. Kyuberis[2], Oleg L. Polyansky[2,3], Nikolai Zobov[2], Robert W. Field[4]

[1]*Physikalisch-Chemisches Institut, Justus-Liebig-Universität Giessen, Heinrich-Buff-Ring 17, 35392 Giessen, Germany.*

[2]*Institute of Applied Physics, Russian Academy of Science, 46 Uljanov Street, Nizhny Novgorod, Russia.*

[3]*Department of Physics and Astronomy, University College London, Gower St, London, UK.*

[4]*Department of Chemistry, Massachusetts Institute of Technology, Cambridge, Massachusetts 02139, USA.*

*e-mail: georg@mellau.de

I. Correspondence principle for the Morse potential

The Morse potential[1]

$$V\left(r\right) = D_e\left(1 - e^{-\beta(r-r_e)}\right)^2$$

is one of the few quantum mechanical systems where the classical and quantum frequencies have analytical solutions. The eigenenergies measured from the potential minimum are

$$E\left(v\right) = \omega_e\left(v + \frac{1}{2}\right) - \frac{\omega_e^2}{4D_e}\left(v + \frac{1}{2}\right)^2$$

and the quantum frequency is

$$\Delta E\left(v''\right) = E\left(v''+1\right) - E\left(v''\right) = \omega_e - 2\left(v''+1\right)\frac{\omega_e^2}{4D_e}$$

$$\Delta E\left(v'\right) = E\left(v'\right) - E\left(v'-1\right) = \omega_e - 2v'\frac{\omega_e^2}{4D_e}$$

where v'' is the quantum number of the lower state and v' is the quantum number of the upper state.

Using the inverse relation between the v quantum number and the $E(v)$ eigenenergies measured from the potential minimum

$$v = \frac{4D_e\omega_e - \omega_e^2 - 4\sqrt{D_e^2\omega_e^2 - E\left(v\right)D_e\omega_e^2}}{2\omega_e^2}$$

we obtain the *eigenenergy dependence* of the quantum frequency

$$\Delta E\left(E''\right) = E' - E'' = \omega_e\left(1 - \frac{E''}{D_e}\right)^{\frac{1}{2}} - \frac{\omega_e^2}{4D_e} \tag{1}$$

$$\Delta E\left(E'\right) = E' - E'' = \omega_e\left(1 - \frac{E'}{D_e}\right)^{\frac{1}{2}} + \frac{\omega_e^2}{4D_e} \tag{2}$$

where E'' is the eigenenergy of the lower state and E' is the eigenenergy of the upper state measured from the potential minimum.

We obtained a quantum formula representing the discrete dependence of the quantum frequency from the lower eigenenergy E'' or upper eigenenergy E'. The discrete dependence is “hard wired” in this quantum mechanical description of the quantum frequency. Equations 1 and 2 are equivalent forms depending on lower/upper *discrete* eigenenergies. The single difference between them is the different definition of the energy axis used to display the energy dependence of the quantum frequency. Out of all such axis definitions, one is very special. The existence of this axis is based on the observation that the Morse potential parameters and eigenenergies fulfill the following ad hoc algebraic equation (it seems that we report this relation for the Morse potential for the first time despite its fundamental importance):

$$E_0' - E_0'' = \omega_e\left(1 - \frac{\frac{\omega_e}{2} + \frac{E_0'+E_0''}{2}}{D_e}\right)^{\frac{1}{2}}. \tag{3}$$

In Equation 3 the eigenenergies $E_0 = E - E(0)$ are given relative to the ground state corresponding to eigenenergies we detect in spectroscopic experiments. The classical oscillation frequency[2] with total energy E in a Morse potential is

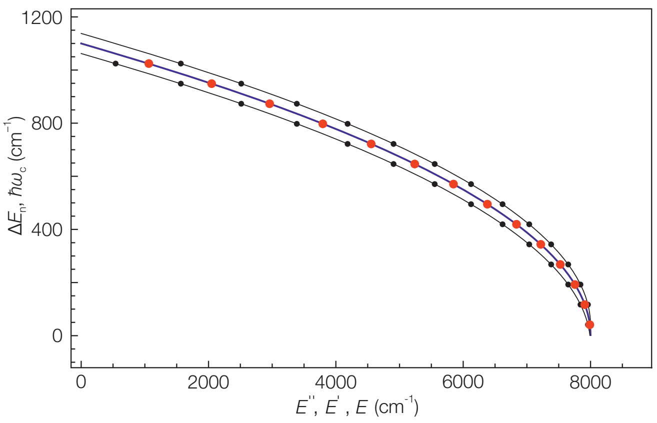

FIG. S1. The $E \longleftrightarrow \frac{\omega_e}{2} + \frac{E_0'+E_0''}{2}$ classical to quantum frequency correspondence for the Morse potential. Black: The $\Delta E_n(E'')$ and $\Delta E_n(E')$ eigenenergy spacings. Red: The $\Delta E\left(\frac{\omega_e}{2} + \frac{E_0'+E_0''}{2}\right)$ eigenenergy spacings. Blue: The classical oscillation frequency with total energy E.

$$\omega_c(E) = \omega_e\left(1 - \frac{E}{D_e}\right)^{\frac{1}{2}}. \tag{4}$$

From the similarity of equations 3 and 4 we observe that for the Morse potential there is an exact classical to quantum mechanical correspondence regarding the $\hbar\omega_c(E) \approx \Delta E_n(E_n)$ relation if we consider the following correspondence between the quantum mechanical eigenenergies and classical total energy

$$E \longleftrightarrow E_n = \frac{\omega_e}{2} + \frac{E_0' + E_0''}{2}. \tag{5}$$

Even if the Morse potential is a fundamentally anharmonic potential the calibration of the mean spectroscopic eigenenergies to the classical oscillation frequencies must be performed based on the ω_e harmonic frequency. This is a very intuitive result: The zero point energy correction has to be evaluated according to the low amplitude classical oscillation frequency. This type of zero point correction is needed in *general* to match the classical and quantum frequencies at high v quantum numbers. In the case of the Morse potential the two frequencies match exactly even for low quantum numbers. For some potentials correspondence $\hbar\omega_c(E) \approx \Delta E_n(E_n)$ is exact or approximately exact even in the energy range corresponding to low quantum numbers.

Based on this correspondence we can consider the set of experimental data points defined

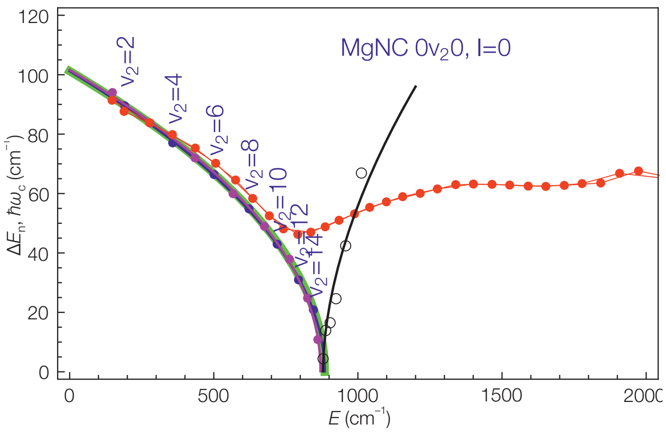

FIG. S2. The quantum and classical frequency for the bending vibrational states for MgNC along the reaction coordinate based on the LIF spectrum[3]. Blue: $\ell = 0$ states. Magenta: $\ell = 1$ states. Red: The *ab initio* $\ell = 0$ and $\ell = 1$ states[4]. Black: Possible assignment of the unassigned lines. Green: The frequency curve for a pure Morse potential with D_e=885 cm^{-1}. The frequency plot shows a pure Morse type dynamics of the Mg-CN bending up to 885 cm^{-1} and suggests the existence of a kink in the potential similar to HNC but much more pronounced. For this plot a few states[3] have been reassigned: v_2=12 line shifted with -2.5 cm^{-1}, v_2=14 line shifted with -1.1 cm^{-1}, v_2=13 line shifted with -1.2 and v_2=15 assigned to the line at 770.4 cm^{-1}.

as

$$\left\{ \frac{\omega_e}{2} + \frac{E_0' + E_0''}{2}, E_0' - E_0'' \right\}. \tag{6}$$

We can extract the harmonic frequency ω_e and the dissociation energy D_e from spectroscopic eigenenergies by fitting these data points to classical oscillations (eq. 4). This fit is performed in a self-consistent iteration with regard to ω_e. The D_e does not have to correspond to a real dissociation energy, the existence of a kink in the potential energy or any other dynamical change can be studied based on equation 4. Figure S2 shows such an example for the bending states of the floppy MgNC radical.

II. Correspondence principle for the symmetric double-well quartic potential

We reformulated the symmetric double well quartic potential

$$V(x) = E_b - Bx^2 + Ax^4 \qquad , B > 0 \tag{7}$$

to a form more suitable for the analysis of the correspondence principle in the neighborhood of a saddle point

$$V(x) = E_b + E_b\left(-2\left(\frac{x}{x_m}\right)^2 + \left(\frac{x}{x_m}\right)^4\right) \tag{8}$$

$$= E_b\left(1 - \left(\frac{x}{x_m}\right)^2\right)^2 \tag{9}$$

$$\omega_0 = \sqrt{\frac{4B}{M}} \qquad E_b = \frac{B^2}{4A} \qquad x_m = \sqrt{\frac{B}{2A}} \quad E_0 = \frac{\hbar\omega_0}{2} \quad m = \frac{2\hbar^2 E_b}{E_0^2 x_m^2} \tag{10}$$

where E_0 harmonic zero point energy in each of the two wells, m the mass of the system. The eigenenergy spectrum E_n for this one-dimensional potential depends only from a single parameter $i = E_b/E_0$. This is equivalent with the $E_n(\sqrt{mE_b}x_m)$ dependence on the system mass and potential parameters. Figure 2 for example displays systems with an overall constant E_0 and x_m and with increasing E_b and m where the mass scales as $m = i \times \frac{2\hbar^2}{E_0 x_m^2}$ with i. Figure 2 is also equivalent with a set of systems with an overall constant mass m and x_m but for each i a different E_b and E_0 where E_0 scales as $E_0 = i \times \frac{2\hbar^2}{m x_m^2}$ with i. A system with any concrete set of (m, E_b, x_m) parameters corresponds to a given i and thus to the family of curves drawn on figures 2,3 and 6; these figures shows the effect of the saddle point for all possible quartic double-well potential systems. The eigenenergies $e_n = E_n/E_0$ within the semiclassical approximation are given for the region below the barrier by the roots of the transcendental equation

$$\frac{4i}{3\pi}\sqrt{\sqrt{\frac{e_n}{i}}+1}\left(E\left(\frac{2}{\sqrt{\frac{i}{e_n}}+1}\right) - \left(1-\sqrt{\frac{e_n}{i}}\right)K\left(\frac{2}{\sqrt{\frac{i}{e_n}}+1}\right)\right) = n + \frac{1}{2}$$

and above the barrier by the roots of the

$$\frac{4\sqrt{2}i}{3\pi}\sqrt[4]{\frac{e_n}{i}}\left(\left(\sqrt{\frac{e_n}{i}}-1\right)K\left(\frac{1}{2}\left(\sqrt{\frac{i}{e_n}}+1\right)\right)+2E\left(\frac{1}{2}\left(\sqrt{\frac{i}{e_n}}+1\right)\right)\right)=n+\frac{1}{2}$$

equation; $E(x)$ is the the complete elliptic integral and $K(x)$ is the complete elliptic integral of the first kind.

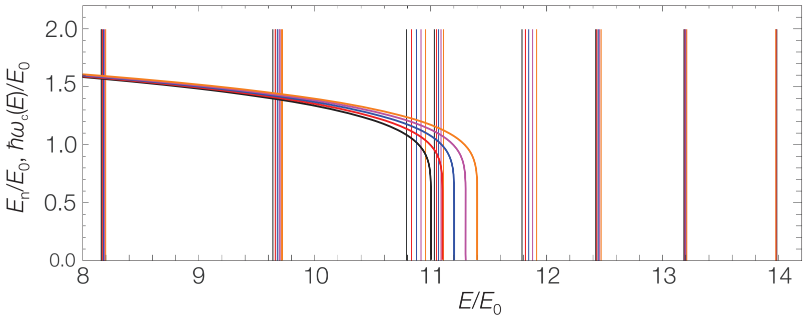

FIG. S3. The eigenenergies and the classical frequencies of the barrier-proximal states for quartic potentials with $E_b = i \times E_0$ with i =11 (black), 11.1 (red), 11.2 (blue), 11.3 (purple), and 11.4 (orange). Increasing the barrier height, eigenenergies in the neighborhood of the saddle point change their position to avoid low quantum frequencies. The barrier-proximal states are more sensitive to changes of the barrier height as is the case for states far below and above the barrier.

Nr	E_n	E	$\Delta E_n(E)$	$\hbar\omega_c(E)$
3	2.916	3.851	1.847	1.848
4	2.916	3.851	1.847	1.848
5	4.762	5.651	1.753	1.755
6	4.762	5.651	1.753	1.755
7	6.516	7.347	1.640	1.644
8	6.516	7.348	1.641	1.644
9	8.156	8.908	1.481	1.502
10	8.156	8.919	1.501	1.501
11	9.636	10.224	1.152	1.296
12	9.658	10.355	1.371	1.263
13	10.788			
14	11.029			
15	11.786	12.116	0.636	0.695
16	12.422	12.815	0.762	0.755
17	13.184	13.596	0.800	0.806
18	13.985	14.421	0.849	0.850
19	14.833	15.290	0.889	0.891

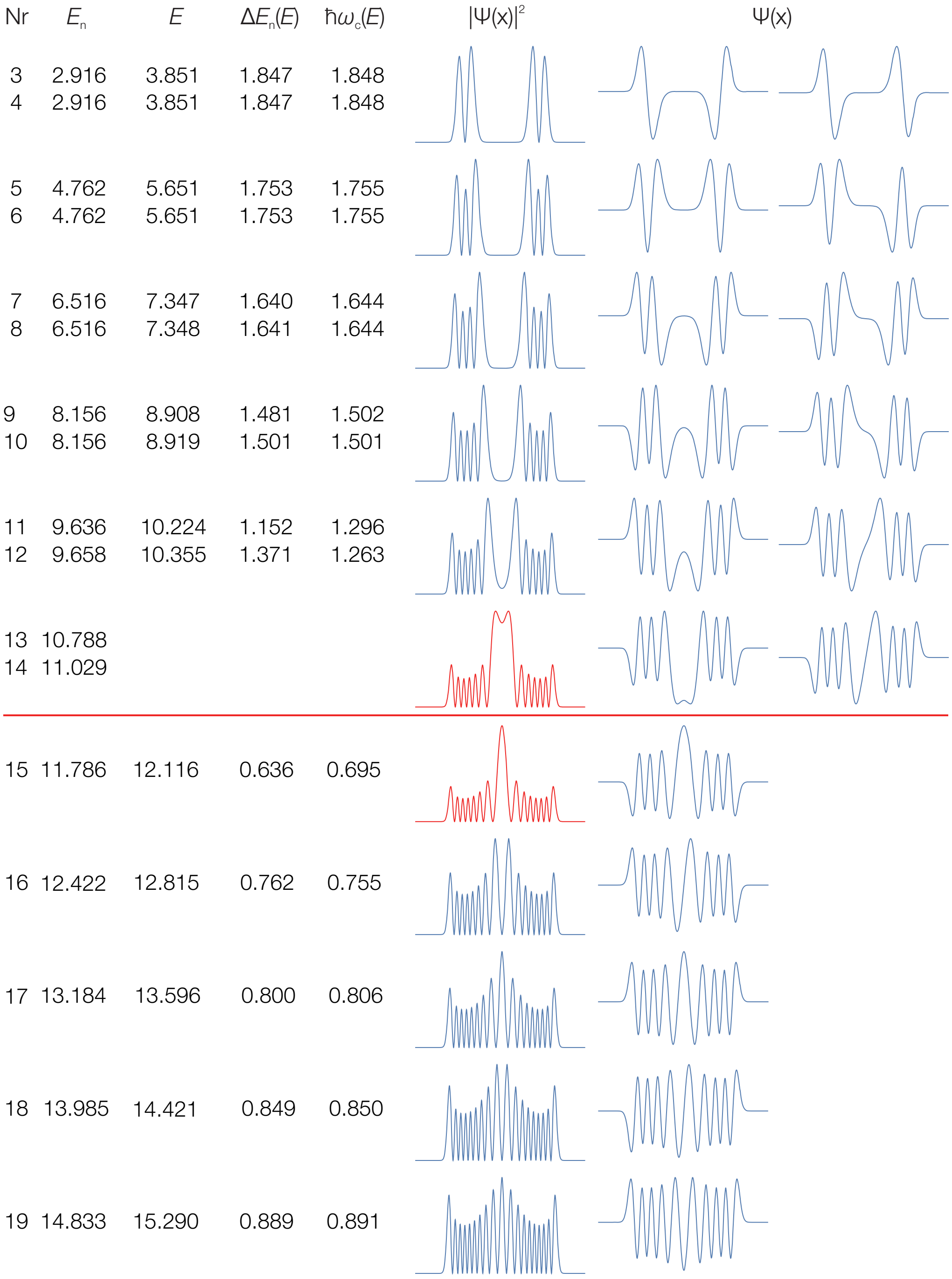

FIG. S4. The quantum frequency analysis of the states in the neighborhood of the saddle point for the symmetric double-well quartic potential with the $E_b = 11 \times E_0$ barrier height.

III. Molecular dynamics at the isomerization barrier: The eigenstate perspective

The models used in the quantum frequency analysis are

$$\omega_c\left(E\right) = \omega_0 \left(1 - \frac{E_0(0) + \frac{E_0'' + E_0'}{2}}{E_b}\right)^{\frac{1}{m}} \tag{11}$$

$$\hbar\omega_l(E) = \frac{\pi E_0 \sqrt{\sqrt{\frac{E-E_b}{E_b}+1}+1}}{K\left(\frac{2}{1+\frac{1}{\sqrt{\frac{E-E_b}{E_b}+1}}}\right)} \tag{12}$$

$$\hbar\omega_u(E) = \frac{\pi E_0 \sqrt[4]{\frac{E-E_b}{E_b}+1}}{\sqrt{2}\times K\left(\frac{1}{2}\left(1+\frac{1}{\sqrt{\frac{E-E_b}{E_b}+1}}\right)\right)}. \tag{13}$$

The quantum frequencies in the neighborhood of the saddle point have a logarithmic dependence $2\pi/\ log(\frac{64E_b}{E_b-E})$. The quantum frequencies of the bond-breaking states in the neighborhood of the saddle point have a universal logarithmic dependence $\pi/\log(\frac{64E_b}{E_b-E})$, the model 13 is much more accurate than in the energy region below the barrier.

TABLE S1. The parameters of the HCN and HNC quantum frequency analysis in cm^{-1}. E_b, m and ω_0 are the parameters of the model 11. The ω_c is the parameter adjusted for the model 12 with the fixed E_b.

State	E_b	m	ω_0	ω_c	E_b(HNC)	m(HNC)	ω_0(HNC)
0,0^{0e},0	16686(17)	8.8(1)	711(1)	793(22)	10986(40)	[12.0]	462(3)
0,0^{0e},1	16217(1)	9.2(2)	708(2)	801(23)	10881(16)	[12.0]	458(1)
0,0^{0e},2	16102(1)	9.3(2)	705(2)	800(20)	10859(38)	[12.0]	452(2)
0,0^{0e},3	15993(1)	10.3(5)	698(4)	803(24)	10722(17)	[11.5]	447(2)
0,0^{0e},4					10806(154)	[12.0]	440(2)
1,0^{0e},0	15910(34)	7.4(4)	700(5)	563(13)	9782(128)	[10.3]	439(2)
2,0^{0e},0	14929(12)	9.0(3)	672(2)	575(4)	8764(24)	[10.3]	427(4)
3,0^{0e},0	13757(1)	11.2(5)	646(3)	748(28)	7317(165)	8.3(11)	410(4)
1,0^{0e},1	15761(1)	10.3(6)	682(5)		9717(63)	[11.5]	429(2)

TABLE S2. The comparison of the barrier heights E_b determined in the quantum frequency analysis with the barrier heights of the one dimensional effective potentials $\Delta V_{(\nu_1,\nu_3)}$.

Nr	State	E_b^{HCN}	$\Delta V_{(\nu_1,\nu_3)}^{HCN}$	ΔE_b^{HCN}	E_b^{HNC}	$\Delta V_{(\nu_1,\nu_3)}^{HNC}$	ΔE_b^{HNC}
1	$0,0^{0e},0$	16686	16344	342	10986	10924	62
2	$0,0^{0e},1$	16217	16241	-24	10881	10881	0
3	$0,0^{0e},2$	16102	16136	-34	10859	10835	24
4	$0,0^{0e},3$	15993	16031	-38	10722	10789	-67
5	$0,0^{0e},4$		15927		10806	10744	62
6	$1,0^{0e},0$	15910	15597	-313	9782	9811	-29
7	$2,0^{0e},0$	14929	14889	40	8764	8774	-10
8	$3,0^{0e},0$	13757	14217	-460		7804	
9	$1,0^{0e},1$	15761	15507	254	9717	9785	000

IV. The quantum frequency analysis of the bond-breaking states

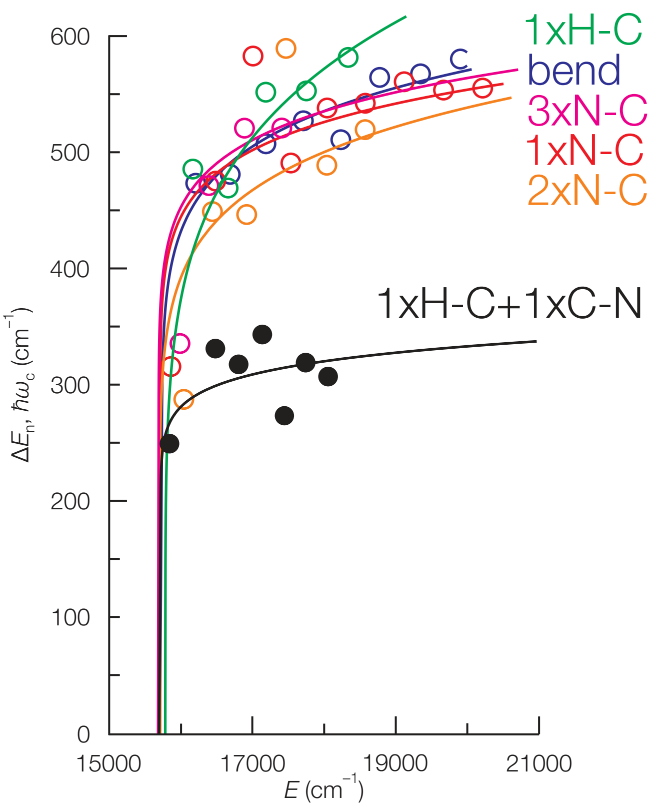

FIG. S5. The quantum frequency analysis of the bond-breaking $H_{0.5}CNH_{0.5}$[5] eigenenergies for different (ν_1, ν_3, ℓ) stretch excitations. For the bend + 1×C-N stretch + 1×H-C stretch the quantum frequency of the bond-breaking states drops significantly in comparison with all other series. The theoretical description within the adiabatic approximation of bond-breaking states is based on the assumption that the stretch quantum numbers remain well-defined for bond-breaking+stretch mixed states. This figure proves that in zero order, all stretch excitations are still well-defined up to very high excitation energies. For each (ν_1, ν_2, ℓ) series the quantum frequency dependence is similar to the one obtained for the model potential.

TABLE S3. The parameters of the quantum frequency analysis for the bond-breaking states in cm^{-1}. E_b, m and ω_0 are the parameters of the model 11. The ω_c is the parameter adjusted for the model 13 with the fixed E_b.

Nr	State	ω_c	m	ω_0	E_b
1	$0,0^{0e},0$	750(14)	[11.0]	655(8)	16684(140)
2	$0,0^{0e},1$	721(20)	12.8(25)	615(20)	16202(5)
3	$0,0^{0e},2$	766(28)	[12.8]	622(9)	16044(2)
3	$0,0^{0e},3$	812(25)	12.0(51)	646(67)	15906(13)
4	$1,0^{0e},0$	1261(27)	9.3(32)	716(68)	15955(6)
5	$2,0^{1e},0$	1115	[6.7]	[756]	15925(1)
6	$1,0^{0e},1$	717(30)	[6.0]	472(23)	

V. The HNC quantum frequency analysis

At a first glance, the HNC quantum frequencies do not show any clear decrease of the quantum frequencies at the barrier. It seems that the eigenenergies cannot be connected to the dynamic isomerization barriers in a similar way as has been done for HCN. The analysis of the HNC quantum frequencies is based on the following assumptions and observations:

a) The saddle point localized states correspond both to HCN and to HNC data sets. It is possible to append the same saddle point localized state to the list of HCN and HNC eigenstates used to set up the quantum frequency lists. The two new quantum frequencies are consistent with either of the two quantum frequency lists and extend both curves in the region of the saddle point.

b) We can impose that the barrier heights resulting from the HNC $0x\nu_3$ quantum frequency analysis for $\nu_3 = 1, 2, 3, 4$ including the localized state to both progressions must match the effective barrier heights[9]. This assumption forces the m parameters of model 11 to a common value for these four series to the ad hoc value of $m = 12$. A similar m value has been kept fixed in all other HNC quantum frequency fits.

c) The vibrational effective Hamiltonian for the HNC well with a kink in the bending potential has been constructed in reference 7. The bending eigenenergies form two disjunctive sets HNC^{I} and HNC^{II} of vibrational states each with its own spectroscopic parameters. The lower set of states has a bigger ω_0 harmonic frequency than the upper set. This may be expected from the visual inspection of the bending potential, the potential in the region of HNC^{II} has a steeper slope than for HNC^{I} resulting in a lower value of the ω_0 parameter at high vibrational excitation. The quantum frequency analysis presented here resembles clearly this double set eigenenergy structure. There are in fact two effective frequency plots as shown in figure S6 corresponding to the upper and lower set of vibrational eigenenergies.

Based on these assumptions it was possible to fit the HNC quantum frequencies, the fits resulted in correct barrier heights for all cases. The effective Hamiltonian which describes both HNC^{I} and HNC^{II} sets has been modeled by adding two Gaussian corrections to equation 11. From this analysis resulted a mean ω_0 parameter. An alternative analysis gives two different ω_0^{I} and ω_0^{II} parameters as shown in figure S6. In both cases we can determine from the eigenstate analysis the inflection point of the HNC effective, adiabatic-

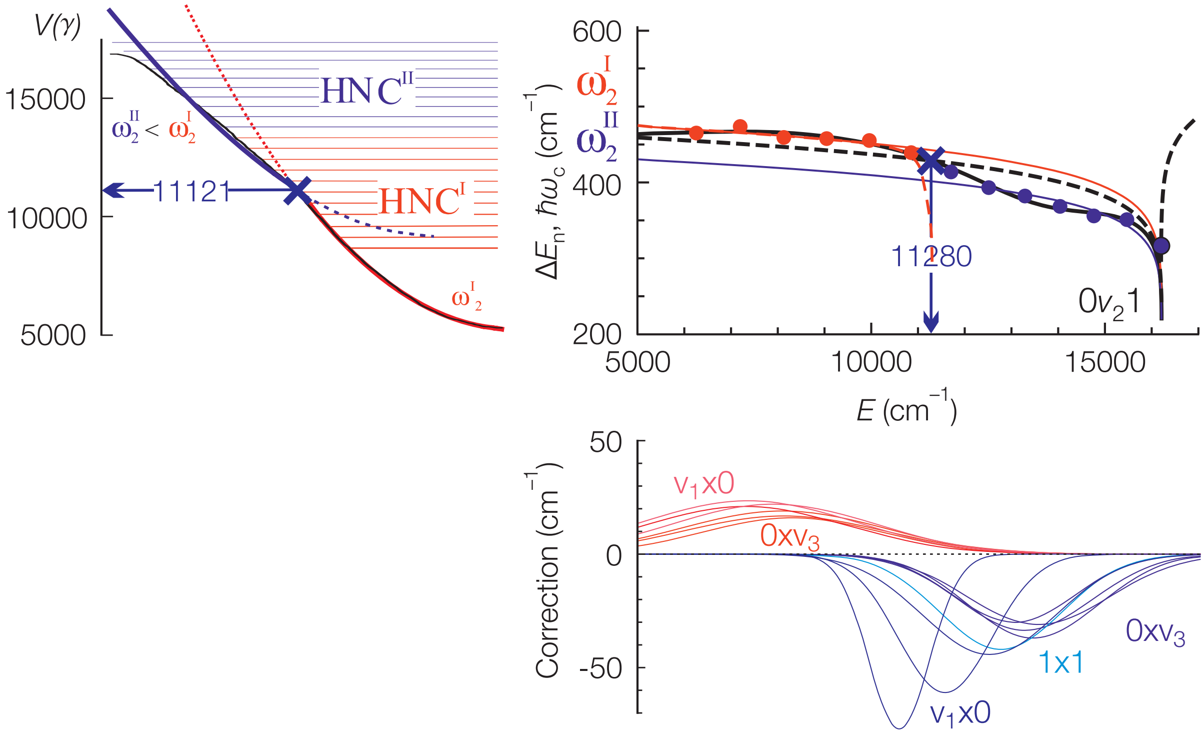

FIG. S6. Upper left and right panel: The isomerization potential and the quantum frequency analysis of the N-C stretch+bending states for HNC. The HNC bending eigenenergies form two disjunctive sets HNC^{I}(red) and HNC^{II}(blue) of vibrational states[7]. The HNC quantum frequency curve shows the pattern of the potential kink allowing to obtain the inflection point from spectroscopic data. Lower panel: The stretch excitation dependent correction needed to remove the effect of the potential kink from the quantum frequency curves.

bend potential as shown in figure S6.

The HNC quantum frequency curves give an alternative description of the spectroscopic pattern resulting from the existence of a potential kink[7]. The kink in the potential forces the eigenenergies of the quantum system to a structure with a similar decrease of the quantum frequency as found for a saddle point. A very pronounced decrease of the quantum frequencies at a potential kink is shown for MgNC in figure S2.

VI. Localization of molecular wavefunctions at the dynamic saddle points

The localization probability of the [H,C,N] saddle point wavefunctions must satisfy the $\Delta p \times \Delta q \geq \frac{\hbar}{2}$ Heisenberg uncertainty relation. This means that there is a minimum width for the localization probability lobe centered at the saddle point for the saddle point localized states. If we assume that the multidimensional wavefunction separates in one-dimensional ones, the highest localization probability in the bending coordinate γ corresponds to the localization probability of a minimum Gaussian wavepacket. These are states where the product $\Delta p \times \Delta q$ is exactly equal to $\hbar/2$ and have a general form wave function

$$\Psi(q) = \frac{1}{(2\pi\Delta Q^2)^{1/4}} e^{\frac{i\langle P\rangle q}{\hbar}} e^{-\left(\frac{q-\langle Q\rangle}{2\Delta Q}\right)^2}$$

and probability density $P(q) = \Psi(q)\Psi^*(q)$ curve

$$P(q) = \Psi(q)\Psi^*(q) = \frac{1}{(2\pi\Delta Q^2)^{1/2}} e^{-\frac{(q-\langle Q\rangle)^2}{2\Delta Q^2}}.$$

The one-dimensional bending probability density curves of the saddle point localized wavefunctions have been fitted at the transition state at $\gamma_{H-CN} = 76°$ with the function

$$C \frac{1}{(2\pi\Delta\gamma^2)^{1/2}} e^{-\frac{(\gamma-\langle\gamma\rangle)^2}{2\Delta\gamma^2}}.$$

The parameters of this analysis for [H,C,N] are given in table S4 and are shown in figure 5.

TABLE S4. Saddle point localized states for the [H,C,N] molecular system. $\langle\gamma\rangle$ is the position of the stretch excitation dependent dynamical saddle point, $\Delta\gamma$ is the width of the localization probability curve.

Nr	State	$\langle\gamma\rangle$	$\Delta\gamma$	C	T(cm^{-1})	ω_l(cm^{-1})
1	$0,26^{0e},0$	78.2(2)	6.5(2)	0.85(2)	15890.0	311
2	$0,26^{0e},1$	78.8(2)	7.0(2)	0.81(2)	17840.0	230
3	$0,26^{0e},2$	78.8(5)	8.6(6)	0.67(3)	19757.9	179
4	$0,26^{0e},3$	80.4(5)	8.6(6)	0.74(4)	21668.1	130
5	$3,24^{0e},0$	77.4(3)	6.2(3)	0.55(2)	22852.9	123
6	$0,25^{1f},0$	78.5(5)	5.0(1)	0.60(1)	15585.1	216
7	$0,27^{1f},2$	79.6(3)	7.2(4)	0.58(2)	20151.9	194
8	$1,25^{1f},0$	74.8(3)	7.7(3)	0.83(3)	18412.0	90
9	$2,25^{1f},0$	74.2(1)	5.5(1)	0.43(1)	20962.7	200

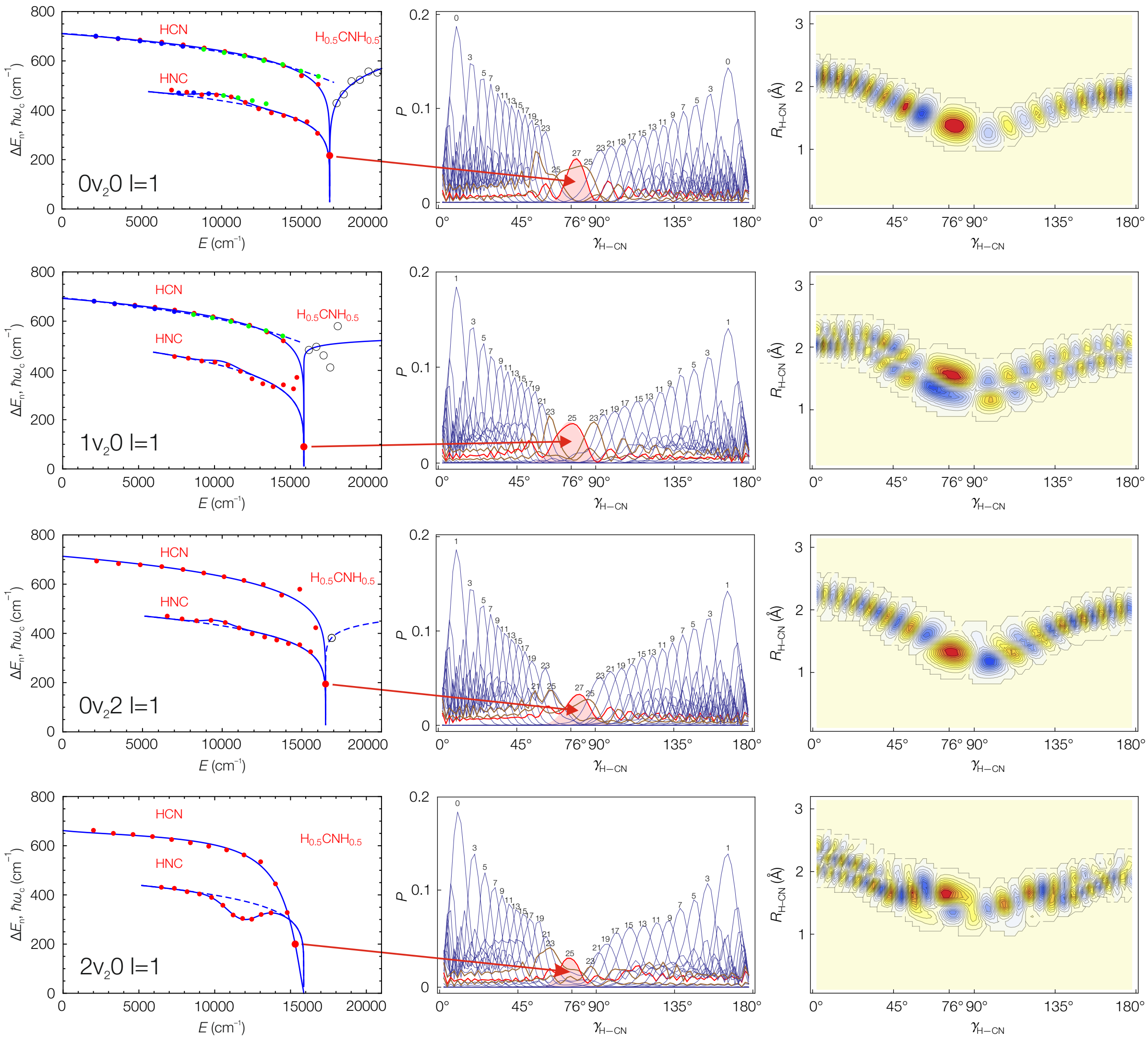

FIG. S7. The emergence of the $J = 1^f$ saddle point localized states for the [H,C,N] molecular system. Left curves: the quantum frequencies for the bending series of states (red: *ab initio* data, blue: measured data, green: predicted from measurements) and the analytical models (blue: equation 11). Middle panels: the one-dimensional projection of the wavefunctions on the bending coordinate (red surface: the fitted minimum Gaussian wavepacket probability density curve). Right panels: the two dimensional projection of the wavefunctions for the saddle point localized states. The one dimensional projections show how the harmonic type, single-well bending wavefunctions of HCN and HNC converge to a final saddle point localized state; these states are spatially localized to a high extent at the transition state at $\gamma_{\text{H-CN}} = 76°$.

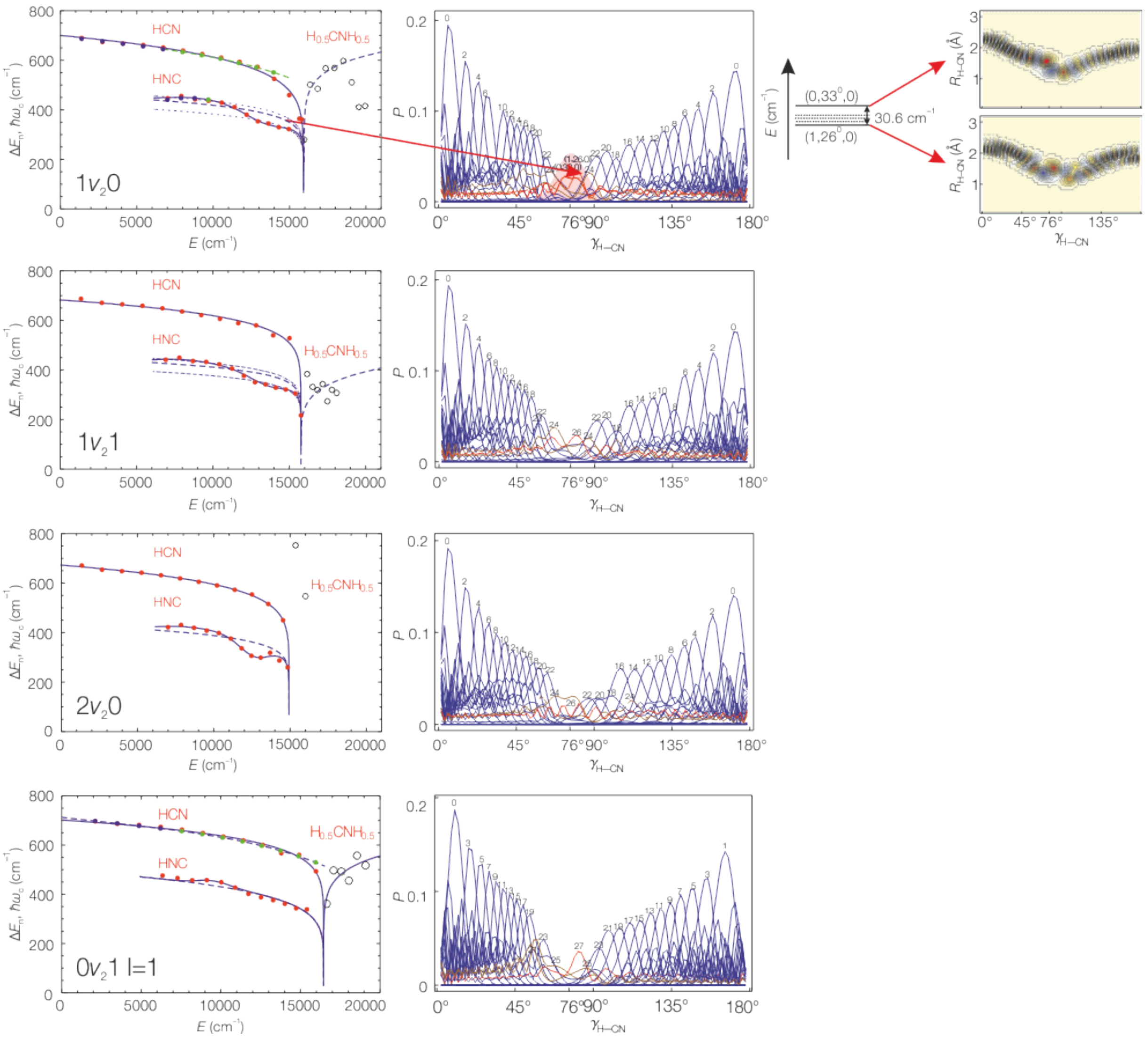

FIG. S8. Examples of (ν_1, ν_3, ℓ) bending series without clear localization of the wavefunction at the saddle point. Either these states mix with other states and loose the localization character or there are simply no eigenstates near the barrier. In the latter case, the quantum frequencies of all states are bigger than the ones found for saddle point localized states. Left curves: The quantum frequencies (red: *ab initio* data, blue: measured data, green: predicted from measurements) and the analytical models (blue: equation 11). Middle panels: the one-dimensional projection of the wavefunctions on the bending coordinate (red surface: the fitted minimum Gaussian wavepacket probability density curve). Right panel: the wavefunctions of the saddle point localized state and of a nearby bond-breaking state.

VII. HCN and HNC data sets

TABLE S5. HCN state term value matrix constants in cm^{-1}, see reference 6 for the definition of the constants. The uncertainty in the last digits (standard deviation) is given in parenthesis. Values that do not have any uncertainty are calculated values. For $v_2 = 14$ the assignment is only tentative, it is based on a few unblended lines.

Nr.	State	$T_{v(J=0)}$	$G_{vz}(v,l)$	B_v	$D_v 10^6$	$H_v 10^{12}$	$q_v 10^3$	$q_{vJ} 10^8$	$q_{vJJ} 10^{12}$	$\rho_v 10^8$	$q_{lv} 10^4$
1	$012^{0}0$	8162.03	8162.03057	1.53232674	3.8211	21.0	9.036	16.16	2.3	-2.75	0.083
2	$012^{2}0$	8178.05	8184.17659	1.53056319	3.7845	19.9					
3	$012^{4}0$	8226.38	8250.90036	1.52731976	3.6748	16.4					
4	$012^{6}0$	8306.77	8361.60706	1.52191403	3.4918	10.6					
5	$012^{8}0$	8418.95	8515.88606	1.51434602	3.2358	2.6					
6	$012^{12}0$	8737.22	8952.10509(12)	1.49268930(76)	3.1094(91)	20.4					
7	$013^{1}0$	8809.89	8811.42903	1.53652626	3.3943	-25.0	9.150	17.50	4.0	-2.07	0.083
8	$013^{3}0$	8842.47	8856.13913	1.53430216	3.3783	-50.0					
9	$013^{5}0$	8907.53	8945.77226	1.52985398	3.3463	-75.0					
10	$013^{7}0$	9004.78	9079.40456	1.52318171	3.2982	-100.0					
11	$013^{9}0$	9134.21	9256.85942	1.51428534	3.2342	-125.0					
12	$013^{11}0$	9295.15	9476.98276	1.50316489	3.1541	-150.0					
13	$014^{0}0$	9443.67	9443.67722	1.54186915	3.0204	10.00	9.234	17.50	4.0	-2.07	0.083
14	$014^{2}0$	9459.53	9465.45261	1.54072392	3.0123	8.0					
15	$013^{13}0$	9487.13	9738.88752(21)	1.49019319(139)	3.0029(18)	-67.8					
16	$014^{4}0$	9508.99	9533.56914	1.53728821	2.9883	6.0					
17	$014^{6}0$	9591.15	9646.09780	1.53156203	2.9483	4.0					
18	$014^{8}0$	9705.74	9803.22258	1.52354538	2.8923	2.0					
19	$014^{10}0$	9852.59	10003.83723	1.51323826	2.8202	-2.0					
20	$014^{12}0$	10031.20	10247.23327	1.50064067	2.7322	-4.0					
21	$014^{14}0$	10241.10	10532.21971(99)	1.48575546(814)	2.9840(117)	-31.0					

TABLE S6. Vibrational expansion constants in cm^{-1} for $H^{12}C^{14}N$. The definition of the parameters can be found in reference 7.

ω_1	3614.47(63)	$y_{1,1,1}$	-15.65(11)	$z_{1,1,1,1}$	-0.008(14)	$z_{1,1,l,l}$	0.0002(39)
ω_2	443.75(13)	$y_{2,2,2}$	-0.380(5)	$z_{2,2,2,2}$	0.0083(2)	$z_{2,2,l,l}$	-0.0062(2)
ω_3	1433.221(33)	$y_{3,3,3}$	19.260(50)	$z_{3,3,3,3}$	-23.824(22)	$z_{3,3,l,l}$	-0.0029(29)
$x_{1,1}$	56.83(43)	$y_{1,1,2}$	-12.73(11)	$z_{1,1,1,2}$	-0.030(23)	$z_{1,2,l,l}$	0.0142(9)
$x_{2,2}$	4.682(48)	$y_{1,2,2}$	0.069(33)	$z_{1,1,1,3}$	32.068(87)	$z_{1,3,l,l}$	0.0215(48)
$x_{3,3}$	291.317(54)	$y_{1,1,3}$	-277.65(29)	$z_{1,1,2,2}$	-0.001(7)	$z_{2,3,l,l}$	0.0053(11)
$x_{1,2}$	10.22(21)	$y_{1,3,3}$	-113.03(12)	$z_{1,1,3,3}$	53.947(64)	$z_{l,l,l,l}$	-0.00008(3)
$x_{1,3}$	448.93(25)	$y_{1,2,3}$	-69.92(13)	$z_{1,1,2,3}$	25.630(79)		
$x_{2,3}$	42.378(51)	$y_{2,3,3}$	-22.419(68)	$z_{1,2,2,2}$	-0.023(1)		
$g_{2,2}$	3.234(20)	$y_{2,2,3}$	0.263(37)	$z_{1,2,2,3}$	-0.039(12)		
		$y_{1,l,l}$	-0.221(14)	$z_{1,2,3,3}$	9.736(45)		
		$y_{2,l,l}$	0.294(4)	$z_{2,2,2,3}$	-0.0069(15)		
		$y_{3,l,l}$	-0.080(14)	$z_{2,2,3,3}$	0.0086(79)		
		$z_{2,3,3,3}$	3.861(24)				

REFERENCES

[1]P. M. Morse, Diatomic molecules according to the wave mechanics. II. Vibrational levels, *Phys. Rev.* **34**, 57–64 (1929)

[2]N. B. Slater, Classical Motion under a Morse Potential, *Nature* **13**, 1352–1353 (1957)

[3]M. Fukushima and T. Ishiwata, The ν_2 bending vibrational structure of the $\tilde{X}^2\Sigma^+$ state of MgNC, *J. Chem. Phys.* **135**, 124311 (2011)

[4]O. Bludsky, V. Spirko, T. Erica Odaka, P. Jensen, and T. Hirano, A theoretical study of the MgNC/MgCN isomerization in the electronic ground state, *J.Mol.Spectrosc.* **13**, 219 - 226 (2004)

[5]G. Ch. Mellau, Rovibrational eigenenergy structure of the [H,C,N] molecular system, *J. Chem. Phys.* **134**, 194302 (2011).

[6]G. Ch. Mellau, Complete experimental rovibrational eigenenergies of HCN up to 6880 cm^{-1} above the ground state, *J. Chem. Phys.* **134**, 234303 (2011).

[7]G. Ch. Mellau, Highly excited rovibrational states of HNC, *J. Mol. Spectrosc.* **269**, 77–85 (2011)

[8]J. H. Baraban, B. Changala, G. Ch. Mellau, J. F. Stanton, A. J. Merer, and R. W. Field, Spectroscopic Characterization of Transition States *Science* **350**, 1338-1342, (2015).

[9]M. Joyeux, S. Y. Grebenshchikov, J. Bredenbeck, R. Schinke, and S. C. Farantos, Intramolecular Dynamics Along Isomerization and Dissociation Pathways *Adv. Chem. Phys.* **136**, 267–303 (2005)
